# Counseling and Psychology Student Experiences of Personal Therapy: A Critical Interpretive Synthesis

**DOI:** 10.3389/fpsyg.2018.01732

**Published:** 2018-09-21

**Authors:** Jane Edwards

**Affiliations:** Madgwick Drive University of New England, Armidale, NSW, Australia

**Keywords:** meta-analysis, trainee therapy student experience, critical interpretive synthesis, personal therapy, counseling training and education

## Abstract

**Background:** Committing to attendance at personal therapy sessions is frequently either mandated or encouraged in many different types of therapeutic trainings across allied health, psychotherapy, social work and counseling. The small number of published accounts have indicated that student experiences of personal therapy can be mixed.

**Methods:** The project examined contemporary interview based research about student experience of personal therapy during training using Critical Interpretive Synthesis method. Ten papers were found which met the search criteria. The papers included a total of 89 participants (75 F).

**Results:** The results comprised 12 themes derived from 89 meaning units gleaned from student experiences presented in the research studies. These were used to inform a synthesizing statement here truncated as follows:

*Beginning therapy is challenging for some students, especially when attendance is mandatory. However, students can experience transformative change by the end of the process, even if they commence the work in a guarded way*.

*Multiple problems can arise in the process of attending therapy as a student, and it can be difficult for a student to know what to do if these challenges are overwhelming*.

*Attending personal therapy brings rich learnings which can be applied in clinical work but can also positively impact learning in the course, and lifelong personal development. The process can enhance the student's understanding of what the client may experience in the therapeutic journey*.

**Conclusions:** Attending personal therapy during training is not a straightforward process for all students. Course leaders and trainers need to be mindful of the possibility that students will struggle with the personal therapy requirement. Course and professional bodies should regularly review personal therapy requirements, being clear about the aims; remaining attuned to the student experience.

## Introduction

The requirement to undergo personal therapy during psychotherapy based training is the focus of multiple research and review papers (for example, Chaturvedi, 2013; Malikiosi-Loizos, 2013; Edwards, 2017; Moertl et al., 2017; So, 2017; McMahon, 2018; Bennett-Levy and Finlay-Jones, 2018). Committing to attendance at personal therapy sessions is an expectation of students in many counseling, allied health, and psychotherapy trainings throughout the world. For example, although the British Association for Counseling and Psychotherapy (BACP) dispensed with the mandatory requirement for personal therapy during training in 2005 (Malikiosi-Loizos, 2013), a review in 2013 showed that 50% of courses still required personal therapy attendance (Chaturvedi, 2013). Similarly the British Psychological Society Division of Counseling Psychology removed specification of required hours in 2015; leaving the mandated participation in personal therapy requirement but no longer referring to a minimum number of hours (Kumari, 2017).

The course or professional association requirement to attend personal therapy sessions ranges from mandatory attendance (Kumari, 2011; Ivey and Waldeck, 2014) to attendance that is optional but encouraged (Digiuni et al., 2013). In a presentation of the historic requirement for trainee engagement with personal therapy, Jacobs (2011) advised that in the early development of the practice of psychoanalysis, “…training analysis had one particular aim, that the trainee should acquire a working knowledge of their unconscious, so that there would be no blind spots when it came to them analyzing their own patient” (p. 428).

There is a strong expectation revealed in multiple theoretical and research papers that attending personal therapy sessions will enhance student learning and, in turn, develop the capacities of the professional body (Von Haenisch, 2011; Edwards, 2017). Personal psychotherapy during training is consistently presented as one way for students to learn about use of the self within therapeutic processes, with a view to positively impacting future therapeutic practice (Kumari, 2011; Von Haenisch, 2011). Trainees are expected to use personal therapy to develop insight about their capacities and limitations, learning to better manage their experiences in interaction with the client. This is achieved through enhancing students' capacity in being able to use a *reflective stance* (Rizq and Target, 2008a), or the engagement of *reflective function* (Ensink et al., 2013).

A range of papers have reflected on problematic aspects of requiring students to attend personal therapy; including that decisions about self-experience requirements in training courses are self-referential, rooted in tradition rather than evidence, and unable to be challenged (Chaturvedi, 2013). The rationale for inclusion of self-development during training is strongly held but weakly conceptualized (Edwards, 2017). Often the student-as-customer environment of the university is not supportive of a self-experience requirement (Edwards, 2013).

Practicing professionals recalling their personal therapy during training, or reflecting on its value as a complement to their current practice, have indicated that the outcomes are not straightforward, and not all found the experience positive. For example, a survey of 95 senior psychiatric trainees across Australia and New Zealand in 2003 found only 22% of respondents considered personal therapy essential (Foulkes, 2003). A survey of 48 psychiatric trainees in London found that one third of respondents had attended personal psychotherapy and reported it as beneficial, and the majority of the remainder indicated they would consider attending psychotherapy in future (Sathanandan and Bull, 2013). Of 25 psychotherapy registrars in the UK who responded to a survey, 15 reported negative effects from their therapy (Macaskill and Macaskill, 1992). These negative effects included psychological distress (29%) and marital or family stress (13%).

The ethics of requiring all students to undertake personal therapy while training as a therapist has received attention. A survey of 170 clinical, and 88 counseling psychologists in Ireland found that more counseling psychologists emphasized the dangers of psychologists working with clients without having undergone a personal therapy experience, and more clinical psychologists questioned the ethics around mandated personal therapy during training (McMahon, 2018).

The principles that underpin student development through personal therapy can be difficult to examine. It is not known to what extent personal therapy enhances trainees' skills, and if these skills are enhanced, how they result in improved outcomes for clients (Bennett-Levy and Finlay-Jones, 2018). There is a challenge to the mandatory nature of the requirement during therapy training, given that the client's motivation for change is key to the outcomes (Malikiosi-Loizos, 2013).

Multiple studies have sought to understand what trainee therapists experience during personal therapy (e.g., Von Haenisch, 2011; Wilson et al., 2015). However, the total number of recent studies available is small, and usually engage survey designs or qualitative method interview-based approaches. Results indicate that for many students the experience of attending personal therapy as part of their training is positive. However, some mixed outcomes have also been reported (Moller et al., 2009; Rizq and Target, 2010; Kumari, 2017).

A Canadian study of 400 psychiatry residents indicated that the personal development opportunity provided through personal psychotherapy positively impacted the confidence of trainees in applying professional skills in practice with their patients (Hadjipavlou et al., 2016). Von Haenisch (2011) reflected that her participants' retrospective accounts of their experience of mandatory psychotherapy during training were similar to those of Rothery (1992) almost two decades prior; that is, with hindsight the participants were convinced of the benefits of attending individual psychotherapy during training even though at the time of their studies many admitted they had been somewhat unwilling to engage and use the opportunity (Von Haenisch, 2011).

This brief introduction and overview of the literature indicates some gaps in what we know about the experiences of students in courses which require or encourage personal development through therapy attendance. Prior work has identified that many training programmes have retained the long-standing personal therapy requirement without a strong rationale or evidence of effective outcomes (Edwards, 2017). Deepening an understanding of students' experience of this requirement is warranted.

### Aim of the research

This study aimed to examine and synthesize documented experiences of students who have attended personal therapy during training. The guiding question for the research was: *what did trainee therapists experience when they attended personal therapy?*

## Method

Following on from the development of meta-analytic review procedures to gain superordinate findings from RCTs and other controlled studies, *meta-synthesis* has gradually emerged as a way to fuse outcomes from multiple qualitative method studies into a higher-order research statement (Edwards and Kaimal, 2016). There are increasing choices of methods available to conduct a meta-synthesis (Thomas and Harden, 2008). Critical interpretative synthesis (CIS) (Dixon-Woods et al., 2006) was chosen to conduct the meta-synthesis reported here. The methodology held the attraction of providing a way to engage the complexity of the topic, and to include criticality as a key driver of the findings.

Critical Interpretive Synthesis (CIS) method was first developed during a literature based study aimed at providing better understanding of the convolution of issues surrounding healthcare access (Dixon-Woods et al., 2006). CIS is a frequently used method of review in healthcare topics, especially reviews in which qualitative method studies are synthesized (Edwards and Kaimal, 2016). *Critical* is included as a descriptor in the method to signal the expectation that researchers will apply deep reflective strategies during the process of the research to ensure that normative assumptions are challenged (Dixon-Woods et al., 2006). Applying CIS method can achieve multiple goals, with the intent to translate existing findings into a new theoretical form (Dixon-Woods et al., 2006). In the review process the researcher examines and interrogates the underlying assumptions represented in the review materials, as well as their own beliefs and values (Dixon-Woods et al., 2006).

### Selection criteria

Papers were included which met the following criteria:

1. Published in a refereed journal
2. Published in English
3. Included qualitative method interview based research with current or former students relevant to relational therapy training programmes such as social work, creative arts therapies, counseling, psychiatry, and clinical psychology.

Only studies which explored experiences of personal therapy located during the time of being a student, whether retrospective interviews with practitioners or research conducted with students during training, were included. Research papers which explored other dimensions such as the impact or effect of personal therapy on quality of practice or other outcomes were not included (e.g., Rake and Paley, 2009). Papers which examined experiences of accessing personal therapy by current professionals were not included (e.g., Orlinsky et al., 2011), except where reflections on experiences as a student were the primary focus. A promising interview based study on motivations for deciding to become a psychotherapist was located (Barnett, 2007). However, although a short discussion on personal therapy appeared in the literature review, the analysis and results did not adequately explore trainee experiences of personal therapy.

It is recognized that personal development can be offered or required in multiple ways during therapy or counseling training. This synthesis focused only on interview-based studies evaluating students' experience of personal therapy. Examples of other types of personal development offerings during training that have been studied, and include; *personal development groups* (for example Payne, 2010), *experiential groups* (e.g., Viljoen and Gildenhuys, 2016), *encounter groups* (e.g., Brison et al., 2015) and *interpersonal therapy training group*s (e.g., Rees and Maclaine, 2016). There is no evidence that any one type of therapeutic offering can optimize personal development opportunities for trainee therapists.

### Search criteria

The search commenced in January 2017 using EBSCO host. The search terms included: trainee “personal therapy,” therapy trainee; student “personal therapy”; mandatory “personal therapy”; personal therapy for counselors. A small number of relevant papers were found this way. Using google scholar to access citations of the papers yielded further papers, as did searching the reference list of each paper for further relevant work.

In undertaking a *first look* at the literature there was no time period indicated. As the number of papers grew beyond what might be usefully managed in the analysis it was decided to limit the time period to the years 2007–2017. However, on close reading of the papers although there were some 20 examples found, many of these did not meet the criteria for inclusion.

Although relevant studies using survey based findings were available (Daw and Joseph, 2007; for example, Bike et al., 2009; Digiuni et al., 2013; Byrne and Shufelt, 2014), only studies which presented findings from in-depth interview data were included. This was intended to ensure that the findings were grounded in personal narratives of students' experiences; with attention to in-depth exploration of students' personal accounts of engaging in therapy during training.

There is no minimum requirement for numbers of papers in a CIS. The goal in seeking papers for the synthesis is to engage adequate papers which can provide what is termed by the founders of CIS a *sampling frame* (Dixon-Woods et al., 2006). As an example, in a CIS study of application of concepts of continuity of care nine papers were included (Heaton et al., 2012). Ten papers were included in a CIS analysis of university students with mental health problems (Markoulakis and Kirsh, 2013).

### Papers included in the CIS study

Following close reading of the 20 papers resulting from the search process 10 papers were found to meet the criteria for inclusion (see Table 1). Two of these papers are presented in the table together (Rizq and Target, 2008a,b) as the two reports use the same student sample. The total number of participants across the papers is 89 (75 F; 14 M). Most of the studies were conducted with trainees or graduates in the United Kingdom (*N* = 7), with the remaining studies conducted with trainees or graduates in Austria, South Africa, and the United States of America.

**Table 1:** Trainee experiences: summary of papers included in the analysis.

**Paper**	**Method**	**Participants**	**Summary of researcher findings**	**Experiences of students presented in the report**
Ciclitira et al., 2012	Biographical-interpretive	19 volunteer counselors (F)	Themes – - personal and professional benefits of personal therapy - challenges of personal therapy, - [understanding] the core ingredients of personal therapy	I am more likely to be able to work with clients' traumatic material and not shut it down as I know these experiences are survivable If I don't engage in personal therapy I could give someone else (client) responsibility for my stuff which could paralyze them Personal therapy helped me to understand the therapist role and how boundaries function I observed some ways that were not helpful (the therapist was too rigid) and I decided that in my own practice I would not be like that Personal therapy helped me understand endings Personal therapy has helped me to understand how even little things can be awkward and uncomfortable for the client There is a process by which it is possible to understand better the role of silence It is helpful to have a boundaried therapist – it is preferred than having a therapist who needs you to know a lot about them It is difficult to find time and money to attend therapy when having care responsibilities at home, studying in the course, and needing to work to cover costs
Ivey and Waldeck, 2014	Unspecified qualitative analysis	9 intern clinical psychologists (7 Female)	Ethical challenges of Mandatory Personal Therapy (MTP) Evolving attitudes toward MPT; Influence of MPT on professional development; Personal impact of MPT; Mutual influence of MPT and clinical training; Relationship between personal therapy and [clinical] supervision.	…we had not been sufficiently informed about the rationale for MPT and had not been given the space to discuss our responses to it Initial stage - … commencing therapy was ‘hard', ‘uncomfortable', and negatively associated with training and assessment Further stage – permeable boundary between the course and personal therapy MPT enhanced understanding of the processes and dynamics of psychotherapy, specifically experiences of transference, interpretation, termination, and therapy technique …personal therapy led to personal growth, self-awareness, increased reflexivity, positive self-regard, and clarification of the role played by the therapist's own issues …personal therapy makes clinical theory salient and, for its part, clinical theory illuminates and heightens appreciation of personal therapy …both personal therapy and the course are ‘hard work' There is vulnerability and emotional exposure that comes with having therapy - this made it difficult to weather the demands of training and the expectation that I be ‘emotionally together' for my own patients …I began to recognize that supervision and personal therapy have distinct roles in practitioner training …there is an ethical dilemma because of the required nature of MPT which may reduce its potency and effectiveness …we concluded that as students it would be hypocritical and ethically problematic to practice psychotherapy without having had personal experience of receiving it
Kumari, 2011	Interpretive phenomenological analysis	8 counseling psychology trainees (7 F)	Experiential learning Personal development The stress of therapy Personal therapy is essential for therapists	The mandatory requirement impacted on the potential to develop a relationship with the therapist, especially in the beginning We learned from the use of techniques by the therapist that were not helpful (e.g. disclosure) I valued “sitting in the other chair” – the experience as client/patient I learned that challenging the client's thinking is OK I appreciated the development of insight – discovering that learning about the self is a lifelong process I found the financial cost of therapy stressful I have to say that going to therapy when there are no obvious problems in your life is challenging I felt pressure having to reach a target number of hours of therapy (40) for qualification I experienced unexpected impacts of therapy attendance such as feeling “in bits” or emotionally overwhelmed which impacted on paid work responsibilities Because we attended mandatory therapy it changed our attitudes to mandatory requirement for therapy attendance – we became more convinced of the need for it It was disappointing that we did not receive information from the course about why personal therapy is needed
Moertl et al., 2017	Grounded theory	10 therapists-in-training at Sigmund Freud Private University (8 F)	Our relationship was so honest and unconditionally supportive at the same time, which allowed me to trust in us and subsequently believe in myself more Our relationship comprised elements of confrontation and limitation, which made me aware of boundaries between us that consequently enabled me to gain a new sense of self-efficacy More awareness of myself and patterns (of relating)	I experienced being seen for who I truly am I experienced the fear of being abandoned by the therapist I felt competitive with other patients I experienced the therapist as reliable and safe – “always there for me” In the beginning I saw the therapist as the expert but as therapy continues it is perceived as a mutual process of walking alongside one another Experiencing the therapist as non-judgemental helped me with life difficulties I brought into sessions I experienced myself as softer and calmer as the therapy came to an end
Rizq and Target (2008a), Rizq and Target (2008b) (complementary papers using the same dataset which present the findings sequentially)	Interpretive phenomenological analysis	9 qualified therapists (6 F)	The self-experiences in personal therapy are intense Professional learning occurs in personal therapy during training and as a practitioner Personal therapy is integral to training	The therapist's willingness to be real and emotionally open encouraged my emotional development Experiences of the analytic Mother/Father can transform our internal world We entered deep water –it is like scuba diving We experienced a sense of immediacy and authenticity which, in turn, can inform therapeutic work I now believe that personal therapy is the only way to understand what the client faces I valued learning about techniques to use and to avoid – learning about the value of the way the therapist provided empathy while understand that the way you provide empathy will be unique. Deciding not to touch patients after an experience of having shoulders massaged by the therapist “ I had to sit with ghastly things” – this assisted in understanding the depth of work needed when clients are severely distressed or wounded The mandatory nature of the requirement could feel like we were “going through the hoops” – “going through some kind of ritual” – just because it is required does not mean it is engaged with integrity There was a significance in being seen – “feeling that connection” The difficulty with being in client role can be embarrassing for us It became more apparent through attending therapy that the therapist needs to know what is happening for them because they use all of their impressions and experiences in the therapy – if they are not consistently developing their self-awareness it is like “going around blind” I know what it is like to be lost in this world – I understand that we need to get through and survive as best we can I experienced that exploring early relationships and early life brought me new insights
Rizq and Target, 2010	Mixed methods – the qualitative arm relevant to the CIS used IPA	12 UK qualified counseling psychologists who had practiced for between 3 and 7 years (9 F)	Emotional safety and control Struggling with ambivalence Self-other boundaries are established	I felt anger and resistance about the mandatory nature of the therapy. This disrupted my ability to engage fully with the therapist (keeping my guard up) I loved experiencing the therapist as surrogate parent, or in parental role It was difficult to have strong emotional reactions to the behavior of the therapist (for example one of the participants described the session as starting with the therapist praying) I felt ongoing sensitivity to power and authority within the relationship – I was not able to say “I am not happy with something” I could not meet the expectations of the therapist – so I asserted a sense of control by saying “I am going to do this on my own terms”
So, 2017	Phenomenology	8 music therapists post training (7 F)	There is a cultural stigma about attending personal therapy in the Asian countries from which the participants originated Personal therapy is a safe place to take difficult feelings – for ex. anger There can be difficulties in therapy – for example feeling pushed	Personal therapy is for me a type of self-care I experienced the start of therapy as “explosive” with expressing or disclosing some things for the first time – it can also be ambiguous and include resistance I felt forced to trust the therapist I thought the session fee was too expensive I tried to be patient with the difficulties I was experiencing – I knew I had to complete a course of therapy to be qualified I valued receiving empathy for the clinical work as well as my status as a foreign student “the therapist blew air into the hose of my diving suit from the top of the ship” “The therapist said – go deeper” My personal therapy was destabilizing and impacted the therapeutic work with clients – “I felt confused and unstable as a therapist” I found it confusing that the therapist spoke in English or (native Asian language) randomly – this made me feel disconnected from the therapist – it was difficult to bring this up with the therapist except toward the end Personal therapy contributed to my healing – for example coming to terms with my experience of traumatic death I believe that receiving empathy from the therapist is powerful – it goes beyond the textbook Personal therapy assisted me in understanding how to use techniques – such as questioning I was able to transfer academic stress that I may have directed toward the professor toward the therapist instead and receive support that the professor in her role may not have been able to give to me Going to therapy is a big step and it is not always easy or possible to tell our family members
Von Haenisch, 2011	IPA	6 psychologists post training with between 1 and 6 years' experience in the field (4 F)	The compulsory aspect of personal therapy has an impact on how it is experienced Personal therapy has impact on personal development Personal therapy has impact on professional development	Personal therapy helped me let go of pain caused in the past It represented the first opportunity in my life to open up about personal issues When starting out in therapy during training it felt like we were “ticking the box” or “jumping through hoops” It offered me the experience of “being on the other side” The therapist showed me how it was possible to be focused, to use intuition, and to show respect It contributed to me better understanding the value of self-care Having concerns about clinical work listened to helped me feel more competent in my clinical practice, as well as the university assignment work I found it easy to see the value of congruence and being non-judgemental The process of personal therapy showed that we need to build the relationship
Wilson et al., 2015	Narrative Analysis	10 female clinical psychologists	The course required attendance at personal therapy There is a stigma to attending personal therapy The therapy process is scary but also exciting Therapy attendance creates the opportunity to be a better therapy practitioner	Mandatory therapy – it was like putting a gun to my head We need to practice what we preach – going into client role is a way to see many dimensions of practice that are not available simply by learning about it or doing it Attending personal therapy helps reduce the “us and them” of mental health care The course referred to personal therapy as *professional development* so I felt that actually using therapy was demeaned by this choice. I started to sleep poorly and I felt anxious and my mood went way down The time period made me feel like we had only just opened up these issues and then we had to pack them away again I tried to change my own therapeutic work based on what worked or didn't in my personal therapy I need to know my vulnerabilities and weaknesses so I don't put them on to my clients.

### Analysis

All of the statements in the papers reporting student self-experience during personal therapy were copied out in full (see the final column in Table 1). These were then broken down further into 89 meaning units revealing multiple dimensions of experiences of students engaging in personal psychotherapy during training. In order to reflect more closely what students experienced during attendance at therapy, examining the meaning units to reveal experiential processes, not just outcomes, was undertaken. For example, the meaning unit “Personal therapy helped me understand endings” was further interrogated through re-reading the research report in the analysis phase. The statement was then re-written to more specifically reflect the student experience as follows: “During sessions I found it hard to think about or work with endings. The therapist supported me to understand how my past experiences contributed to this” (see final column Table 2). The process of analysis was inductive whereby the materials for the analysis were drawn from the data rather than conceived *a priori*. The analysis involved moving back and forth between the synthesizing statements, reports of student statements presented in Table 1, and the original papers. The process required comparing concepts between papers, and describing in detail student experiences of personal therapy represented in the papers.

**Table 2:** Themes and the meaning units which informed them.

Encounters between the therapist and the trainee experienced as difficult cannot easily be addressed	I noticed how even little things the therapist did could be awkward and uncomfortable for me It was difficult to have strong emotional reactions to the behavior of the therapist (for example one of the participants described the session as starting with the therapist praying) I felt ongoing sensitivity to power and authority within the relationship – I was not able to say “I am not happy with something” I could not meet the expectations of the therapist – so I asserted a sense of control by saying “I am going to do this on my own terms” I found it confusing that the therapist spoke in English or (native Asian language) randomly – this made me feel disconnected from the therapist – it was difficult to bring this up with the therapist except toward the end I felt forced to trust the therapist I tried to be patient with the difficulties I experienced – I knew I had to complete a course of therapy to be qualified
Attending personal therapy was problematic and the course didn't help	It was disappointing that we did not receive information from the course about why personal therapy is needed It was difficult to find time and money to attend therapy with my care responsibilities at home, studying in the course, and needing to work to cover costs …we had not been sufficiently informed about the rationale for MPT by the course and had not been given the space to discuss our responses to it The course referred to personal therapy as professional development so I felt that actually using therapy was demeaned by this choice of words …there is an ethical dilemma because of the required nature of MPT which may reduce its potency and effectiveness I have to say that going to therapy when there are no obvious problems in your life is challenging I felt pressure having to reach a target number of hours of therapy (40) for qualification Going to therapy is a big step and it is not always easy or possible to tell our family members The difficulty with being in client role can be embarrassing for us I thought the session fee was too expensive I found the financial cost of therapy stressful
The support of the therapist was valued and transformative	I loved experiencing the therapist as surrogate parent, or in parental role Experiences of the analytic Mother/Father can transform our internal world “the therapist blew air into the hose of my diving suit from the top of the ship” Personal therapy contributed to my healing – for example coming to terms with my experience of traumatic death I experienced the therapist as reliable and safe – “always there for me” Experiencing the therapist as non-judgemental helped me with life difficulties I brought into sessions There was a significance in being seen by the therapist – “feeling that connection” The therapist's willingness to be real and emotionally open encouraged my emotional development Personal therapy helped me let go of pain caused in the past
Personal therapy made it possible to be better informed about client role, and what the client experiences	We need to practice what we preach – going into client role is a way to see many dimensions of practice that are not available simply by learning about it or doing it Attending personal therapy helps reduce the “us and them” of mental health care I now believe that personal therapy is the only way to understand what the client faces We experienced a sense of immediacy and authenticity which, in turn, can inform therapeutic work …we concluded that as students it would be hypocritical and ethically problematic to practice psychotherapy without having had personal experience of receiving it I felt competitive with other patients
Personal therapy negatively impacted university, clinical work and other responsibilities	My personal therapy was destabilizing and impacted the therapeutic work with clients – “I felt confused and unstable as a therapist” Because personal therapy exposed me and made me emotionally vulnerable this impacted on my participation in the course Because personal therapy exposed me and made me emotionally vulnerable this impacted on my work with clients I started to sleep poorly and I felt anxious and my mood went way down I experienced unexpected impacts of therapy attendance such as feeling “in bits” or emotionally overwhelmed which impacted on paid work responsibilities.
It was hard work but also valuable to work at a deep level	“The therapist said – go deeper” We entered deep water –it is like scuba diving I went to some difficult places with the therapeutic work …personal therapy is ‘hard work' “I had to sit with ghastly things” – this assisted in understanding the depth of work needed when clients are severely distressed or wounded
Personal therapy was helpful in managing university and clinical work	Having concerns about clinical work listened to helped me feel more competent in my clinical practice, as well as the university assignment work I was able to transfer academic stress that I may have directed toward the professor toward the therapist instead and receive support that the professor in her role may not have been able to give to me I tried to change my own therapeutic work based on what worked or didn't in my personal therapy I valued receiving empathy from the therapist for the challenges in my clinical work as well as my status as a foreign student
The start of therapy was explosive or guarded	In the initial stage - … commencing therapy for me was ‘hard', ‘uncomfortable', and negatively associated with training and assessment The mandatory requirement impacted on the potential to develop a relationship with the therapist, especially in the beginning Asking me to attend mandatory therapy – it was like putting a gun to my head When starting out in therapy during training it felt like we were “ticking the box” or “jumping through hoops” The mandatory nature of the requirement could feel like we were “going through the hoops” – “going through some kind of ritual” – just because it is required does not mean it is engaged with integrity I felt anger and resistance about the mandatory nature of the therapy. This disrupted my ability to engage fully with the therapist (keeping my guard up) I experienced the start of therapy as “explosive” with expressing or disclosing some things for the first time – it can also be ambiguous and include resistance
Experiences at the beginning and ending of therapy were different	In a further stage of attending therapy – I experienced a more permeable boundary between the course and personal therapy Because we attended mandatory therapy it changed our attitudes to mandatory requirement for therapy attendance – we became more convinced of the need for it In the beginning I saw the therapist as the expert but as therapy continues it is perceived as a mutual process of walking alongside one another I experienced myself as softer and calmer as the therapy came to an end
The need for self-knowledge of the therapist was heightened	It became more apparent through attending therapy that the therapist needs to know what is happening for them because they use all of their impressions and experiences in the therapy – if they are not consistently developing their self-awareness it is like “going around blind” I realized I needed the capacity to work with clients' traumatic material I began to be aware of a huge responsibility around knowing about my own stuff through personal therapy so that I didn't unwittingly paralyze clients with it I need to know my vulnerabilities and weaknesses so I don't put them on to my clients
Learnings from observing the things the therapist did that were ineffective	I observed some ways that were not helpful (the therapist was too rigid) and I decided that in my own practice I would not be like that I found that it was helpful to have a boundaried therapist – my previous therapist told me a lot about herself and I didn't find it useful I valued learning about techniques to use and to avoid – learning about the value of the way the therapist provided empathy while understand that the way you provide empathy will be unique. Deciding not to touch patients after an experience of having shoulders massaged by the therapist We learned from the use of techniques by the therapist that were not helpful (e.g. disclosure)
Enhanced learnings about the therapeutic role and techniques from the experience of the client; beyond the textbook	MPT enhanced my understanding of the processes and dynamics of psychotherapy, specifically experiences of transference, interpretation, termination, and therapy technique I saw first-hand how the therapist role and boundaries need to function …I began to recognize that supervision and personal therapy have distinct roles in practitioner training The process of personal therapy showed that we need to build the relationship I found it easy to see the value of congruence and being non-judgemental Personal therapy assisted me in understanding how to use techniques–such as questioning The therapist showed me how it was possible to be focused, to use intuition, and to show respect It offered me the experience of “being on the other side” I valued “sitting in the other chair”–the experience as client/patient I appreciated the development of insight–discovering that learning about the self is a lifelong process Personal therapy is for me a type of self-care I believe that receiving empathy from the therapist is powerful – it goes beyond the textbook Personal therapy contributed to me better understanding the value of self-care During sessions I found it hard to think about or work with endings. The therapist supported me to understand how my past experiences contributed to this I experienced that exploring early relationships and early life brought me new insights Through experiences in attending the sessions I began to be able to manage the role of silence …personal therapy makes clinical theory salient and, for its part, clinical theory illuminates and heightens appreciation of personal therapy …personal therapy led to personal growth, self-awareness, increased reflexivity, positive self-regard, and clarification of the role played by the therapist's own issues I learned that challenging the client's thinking is OK “I had to sit with ghastly things” – this assisted in understanding the depth of work needed when clients are severely distressed or wounded It represented the first opportunity in my life to open up about personal issues I experienced being seen for who I truly am I experienced the fear of being abandoned by the therapist I know what it is like to be lost in this world – I understand that we need to get through and survive as best we can The time limited period of attendance made me feel like we had only just opened up these issues and then we had to pack them away again

In the first stage of the analysis meaning units were generated through extracting the key statements about student experiences of personal therapy from each of the papers (see Table 1). These meaning units were then gradually corralled into groups by reflecting on statements which appeared similar. The steps taken were to; (1) Read the statements over and over, (2) Find and explore similarities between the statements, and (3) Group similar statements together. The groupings were constantly refined by returning to the original paper, and by comparing the statements in each of these groupings or categories. Once there were multiple statements grouped together the group was tentatively titled with reference to the predominant theme emerging from the statements. Each title was then further refined as more items were added (see column 1 in Table 2). Each title reflected a theme that responded to the guiding question for the synthesis: *what did trainee therapists experience when they attended personal therapy?*

## Results

The following 12 themes were identified through the process of analysis. In the first instance they were group according to positive and negative experiences:

### Positive experiences

- The support of the therapist was valued and transformative
- Personal therapy made it possible to be better informed about client role, and what the client experiences
- It was hard work but also valuable to work at a deep level
- Personal therapy was helpful in managing university and clinical work
- Experiences at the beginning and ending of therapy were different
- The need for self-knowledge of the therapist was heightened
- Learnings from observing the things the therapist did that were ineffective
- Enhanced learnings about the therapeutic role and techniques from the experience of the client; beyond the textbook

### Negative experiences

- The start of therapy was explosive or guarded
- Personal therapy negatively impacted university, clinical work and other responsibilities
- Attending personal therapy was problematic and the course didn't help
- Encounters between the therapist and the trainee experienced as difficult cannot easily be addressed

Critical reflection fundamental to the CIS process was then undertaken by a. Further reflection on the 12 statements generated through the synthesis, b. Writing out responses to the statements, and c. Seeking further literature relevant to personal psychotherapy during training. For example, I initially wrote responses to the statements about personal therapy during training which were the most confronting for me. I felt especially strongly about student experiences of inadequate therapy, or of therapist behavior that was problematic, so this was the first topic addressed in the reflective writing tasks. I also felt sympathy for students who had difficulties in therapy and did not know what to do. I wondered if I had been adequately sympathetic on the very rare occasions when students came to me as a training director to discuss concerns about the personal therapy requirement. When reflecting on the behavior of therapists who engaged in actions the students found difficult, such as being too controlling, giving a shoulder massage, or starting the session with prayer, I explored feeling of being annoyed and astounded. I worried that students in programmes I had led might have experienced these types of interactions and not felt able to come to me or another team member with their concerns. As a response I sought out literature discussing therapists boundaries and role (e.g., Parry and Simpson, 2016), and found other related literature about student participation in personal therapy; some of which was then included in the literature review above (Malikiosi-Loizos, 2013; for example, Kumari, 2017). I reflected on enjoying some of the images of therapy, especially the diving analogy in the paper by So (2017), and wrote creative responses to these pleasurable descriptions.

The process described above resulted in a final *synthesizing argument* presented below, and elaborated in the following discussion section.

Beginning therapy is challenging for some students, especially when attendance is mandatory. However, students can experience transformative change by the end of the process, even if they commence the work in a guarded way.

Multiple problems can arise in the process of attending therapy as a student, and it can be difficult for a student to know what to do if these challenges are overwhelming. When negative impacts are experienced it is not always clear that the university staff are available to help. Some students can feel unable to provide competent clinical work because of the emotional intensity of their personal therapeutic work which they need time and space to process.

Attending personal therapy brings rich learnings which can be applied in clinical work but can also positively impact learning in the course, and lifelong personal development. Some of the processes experienced in personal therapy are completely new, and others seem familiar from course learnings; bringing textbook materials to life. The process can enhance the student's understanding of what the client may experience in the therapeutic journey.

## Discussion

The positive experiences reported in the CIS align with Kumari's (2017) description of positive outcomes from attending personal therapy during training. Personal therapy (1) Enhances students' understanding about their profession through personal experience/learning, (2) Allows students to explore any previously repressed issues, (3) Permits first-hand experience of clinical techniques, (4) Supports greater awareness of what it feels like to be the client leading to greater empathy with their client's challenges, (5) Improves comprehension of interpersonal dynamics which can then increase students' understanding of the aims of therapy, reducing the likelihood of transference reactions, and 6. Improves the trainee's emotional and intellectual functioning (Kumari, 2017).

In Kumari's earlier study (2011) she found that students experienced stress when attending personal therapy, some of which was caused by not at first realizing the personal therapy was mandatory, and then struggling to meet the financial expectation. Trainees also indicated that personal therapy was disruptive to their clinical work because it preoccupied them with their own issues (Kumari, 2011). Trainees could not give their clients full attention, and reported a negative effect on their personal functioning (Kumari, 2011). Some of the negative experiences reported in the CIS undertaken here also covered these aspects. However, there were additional negative issues reported in the further papers synthesized for the CIS, including that if interpersonal challenges arose between the trainee and therapist they could be difficult to address (Rizq and Target, 2010).

This CIS review reported that there is a lack of clarity about expectations as to the purpose and value of personal therapy by students, courses, and by professional bodies. There is a tacit understanding that personal therapy attended for training purposes and, by comparison, personal therapy sought out because of personal distress are not one and the same (Jacobs, 2011). However, the problematic belief that personal therapy for trainees would probably provide the same process and outcomes is evident (Rizq, 2011; Malikiosi-Loizos, 2013).

Student interview feedback about the experience indicated that the course and professional associations could do more to review and research the phenomenon of personal therapy during training. The personal therapy requirement needs a clear aim, and the work undertaken by students needs to meet these aims. Additionally, information given to candidates seems to be either given or heard as *you have no choice you have to do this* rather than a more enlightened communication process by which students are encouraged to ask questions and seek further information.

There is a concern about the lack of interest in the negative aspects of attending therapy during training (Malikiosi-Loizos, 2013; Kumari, 2017). In the absence of a critical, balanced viewpoint, unconscious bias may be present that continually reinforces the need for personal development of trainees to be achieved through personal therapy attendance (Kumari, 2017).

As an educator it was concerning to read that some students who were not happy with their therapist seemed to have no strategies about how to seek information as to whether the therapy they were attending was provided competently and had the potential to be helpful. It was also concerning that despite decades of research in teaching and practice the author had never previously heard of prayer used during counseling or psychotherapy sessions (Rizq and Target, 2010). In searching for further information it was found that there is an established literature about the ethics of pastoral counseling (Zust et al., 2017), and also advice in using prayer in this work (e.g., Weld and Eriksen, 2007). However, even if it was common for the practitioner or (perhaps) the Christian-based service to use prayer in sessions, the fact that the student did not like it raises questions as to why it was not possible to negotiate with the therapist, or change over to a service that was not faith-based.

Student learning gained through reflecting on ineffective strategies of the therapist does not seem an optimal use of therapeutic space, and time. Some examples were; the therapist providing a shoulder massage (Rizq and Target, 2008a), and disclosing extensive personal information (Kumari, 2011; Ciclitira et al., 2012). Therapist behavior described by the student which went beyond the scope of practice, or violated widely held norms about professional conduct, seemed to have occurred without consequence in the trainee's accounts. At the same time it is useful to reflect on a review of 10 years of complaints brought against BACP accredited practitioners which found that 18% of complainants were trainees (Khele et al., 2008).

Although there were many positive opportunities and rich learnings gained through personal therapy there were also some reported challenges. Addressing these is not easy or straightforward. However, therapy professions must take at least some responsibility for the negative as well as positive dimensions of what is offered during training with reference to the student experience.

## Limitations of the study

The process of CIS is not intended to achieve consensus across the research outcomes included from the various studies, but rather present a synthesizing statement that accounts for all of the findings. The trustworthiness of the outcomes is reliant on the procedures used to undertake the analysis and present the findings. Therefore, although informative and helpful for associations and training courses in developing their requirements, the direct applicability is moderate.

Each of the included studies involved a cohort of respondents with higher female numbers than male. Two studies included only female participants (Ciclitira et al., 2012; Wilson et al., 2015). Of the 89 interviewees across the studies, 18% were male reflecting statistics reported about the gender balance in some fields of psychotherapy (for example, Robinson et al., 2017). This gender ratio also reflects the male-female make up of respondents in surveys of therapy trainees (for example, Owen et al., 2016). However, there is no way to find out the gender balance in therapy training courses for the review period. Therefore, the absence of gender balance in the respondents reported for the studies could be considered a limitation.

## Conclusion and author recommendations

The findings of this synthesis clearly indicate that attending personal therapy brings rich learnings that can be applied to clinical work in training and future professional practice. This growth opportunity can positively impact engagement in the training course, along with making a positive contribution to lifelong personal development. The process of attending personal therapy can enhance the student's understanding of the client's experience.

The synthesis also revealed that attending personal therapy during training is not a straightforward process for all students. Some students may need other options than attending one-to-one personal therapy to be available to meet course and professional association requirements. Course and professional bodies need to regularly review the evidence of value for the client of the trainee attending personal therapy. Staying attuned to the student experience is key. Students need a clear pathway to follow if they have concerns about the behavior of the therapist, or reject the requirement to continue with further therapy.

It is recommended, from the perspective of the author that courses must do more to engage with students in advance about the need for personal therapy. Course teams should provide information about the options available if therapy is perceived as difficult, persecuting, or negatively impacting on everyday life; what McMahon (2018) described as providing “consideration and protection” (p. 424) if therapy is not helpful. This information must be provided in written form, but also in face to face discussions about the experience, including potential value and benefits along with advice on how to handle difficulties. Providing a third party independent of the course with whom to discuss any issues arising about the value of therapy may support better outcomes for students who have difficulties.

There is ongoing need for further study about the benefits intended to be achieved by personal therapy, and the options available if personal therapy attendance is experienced by the student as not helpful. The effects of attending personal therapy on future competence requires information based in solid, well-conceived studies that deliver plain evidence.

## Author contributions

The author confirms being the sole contributor of this work and approved it for publication.

### Conflict of interest statement

The author declares that the research was conducted in the absence of any commercial or financial relationships that could be construed as a potential conflict of interest.
